# Influence of patient engagement technology on reported perioperative experiences of patients undergoing lung resection

**DOI:** 10.1016/j.xjon.2025.09.044

**Published:** 2025-10-13

**Authors:** Frank Gleason, Benjamin Wei, James Donahue

**Affiliations:** a Division of Cardiothoracic Surgery, Department of Surgery, University of Alabama at Birmingham, Birmingham, Ala; b Birmingham Veterans Administration Medical Center, Birmingham, Ala

**Keywords:** Lung resection, web-based app, perioperative, patient-reported outcomes

## Abstract

**Objectives:**

Patient engagement technologies (PETs) are web-based platforms that provide a means to collect patient-reported outcomes (PROs) as well as guide patients through their surgical journey. The optimal method for collecting PROs is unknown. PROs are an important measure of health care quality We sought to describe the experience of patients undergoing lung resection at our institution using an app-based PET platform.

**Methods:**

Patients undergoing elective lung resection surgery from 2019 to 2023 who enrolled with a PET were identified. Patients received educational content; health checks; and surveys, including the Patient-Reported Outcomes Measurement Information System Global-10 and EuroQOL 5 Dimension surveys. Descriptive statistics were employed to determine utilization and initial observations.

**Results:**

We enrolled 952 patients who underwent lung resection, of whom 88% (838 out of 952) activated the PET and completed the setup survey. More than half (436 out of 838) of patients were women and 68% (229 out of 335) had adequate health literacy. Preoperative Patient-Reported Outcomes Measurement Information System Global-10 and EuroQOL 5 Dimension surveys were completed by 73% (613 out of 838), whereas 37% (309 out of 838) completed an inpatient health check and 39% (325 out of 838) completed a 1-month follow-up survey. Overall, 91% (179 out of 196) reported the PET improved their confidence in postoperative self-care and as a result 86% (169 out of 196) reported feeling less worried about their surgical journey. Use of the PET allowed 39% (77 out of 196) to avoid telephone calls to the hospital care team and 7.6% (15 out of 196) avoided emergency room visits.

**Conclusions:**

Patient engagement technologies provide a way to collect PROs. Among patients who utilize PETs in their perioperative care, there is a reported reduction in telephone calls to providers and emergency room visits, promotion of empowerment in self-care, and reduction of anxiety.

**Figure:**
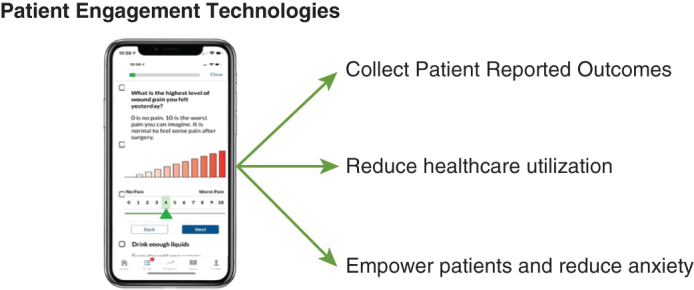
The influence of patient engagement technologies on perioperative care.

**Central Message**

Patient engagement technologies provide a way to collect patient reported outcomes, can reduce health care utilization, empower patients in self-care, and relieve anxiety.

**Perspective**

This is a 5-year retrospective cohort study examining survey completion rates, health care utilization, and patient-reported outcome surveys in patients undergoing lung resection using a web-based application. The use of the app prevented more than 100 telephone calls to providers and 24 emergency room visits. This resulted in improved confidence in self-care and feeling less worried in >85%.

The traditional measures of surgical quality in morbidity, mortality, and readmissions lack insight into the patient experience and perspective. The use of patient reported outcomes (PROs) expands the understanding of the patient perioperative journey and can have a positive influence on care delivered. When counseling patients on expectations after lung resection, PROs collected by Heiden and colleagues^1^ suggest postoperative pain and physical limitations will exist for 6 months, whereas dyspnea may linger for more than a year. Relaying expected postoperative symptom severity and duration to patients could help alleviate postoperative anxiety or frustration related to their recovery.

Various mechanisms for and settings in which this data is obtained exist. A focus on patient satisfaction while minimizing the work burden placed on providers is key. Many advocate for patient engagement technologies (PETs) because they have continuously been associated with high satisfaction rates and lower rates of error in completion compared with paper surveys.^2^, ^3^, ^4^, ^5^ Almost all adults in the United States have access to a cellular phone, which can provide access to PETs and potentially help bridge gaps to access in care.^6^ Patient use of PETs has also been so shown to influence patient health care utilization. Multiple studies report a reduction in care team telephone calls and emergency room (ER) visits across various subspecialties.^7^^,^^8^ However, his has not yet been shown in thoracic surgery patients.

Within thoracic surgery, it remains unclear if PETs are reliable tools for collecting PROs and if they can impact healthcare utilization. We sought to characterize the experience of patients undergoing lung resection using an app-based PET platform.

## Methods

This is a retrospective cohort study evaluating patients undergoing elective lung resection at a tertiary care center from June 2019 to December 2023 who enrolled with a PET (SeamlessMD). All English-speaking patients aged 18 years or older with access to the internet via smartphone, tablet, or computer undergoing elective thoracic surgery were offered enrollment at no charge during their preoperative clinic visit. Various features and functionalities of the PET were discussed with the patient and if they agreed, they were enrolled by a trained clinic nurse. Instructions were provided for downloading, activating, and using the program. Informed consent for participation in this study and publication of data was obtained in clinic. Patients who required emergency surgery, had their elective operation canceled, and those who were inpatient consultations were excluded.

**Figure E1:**
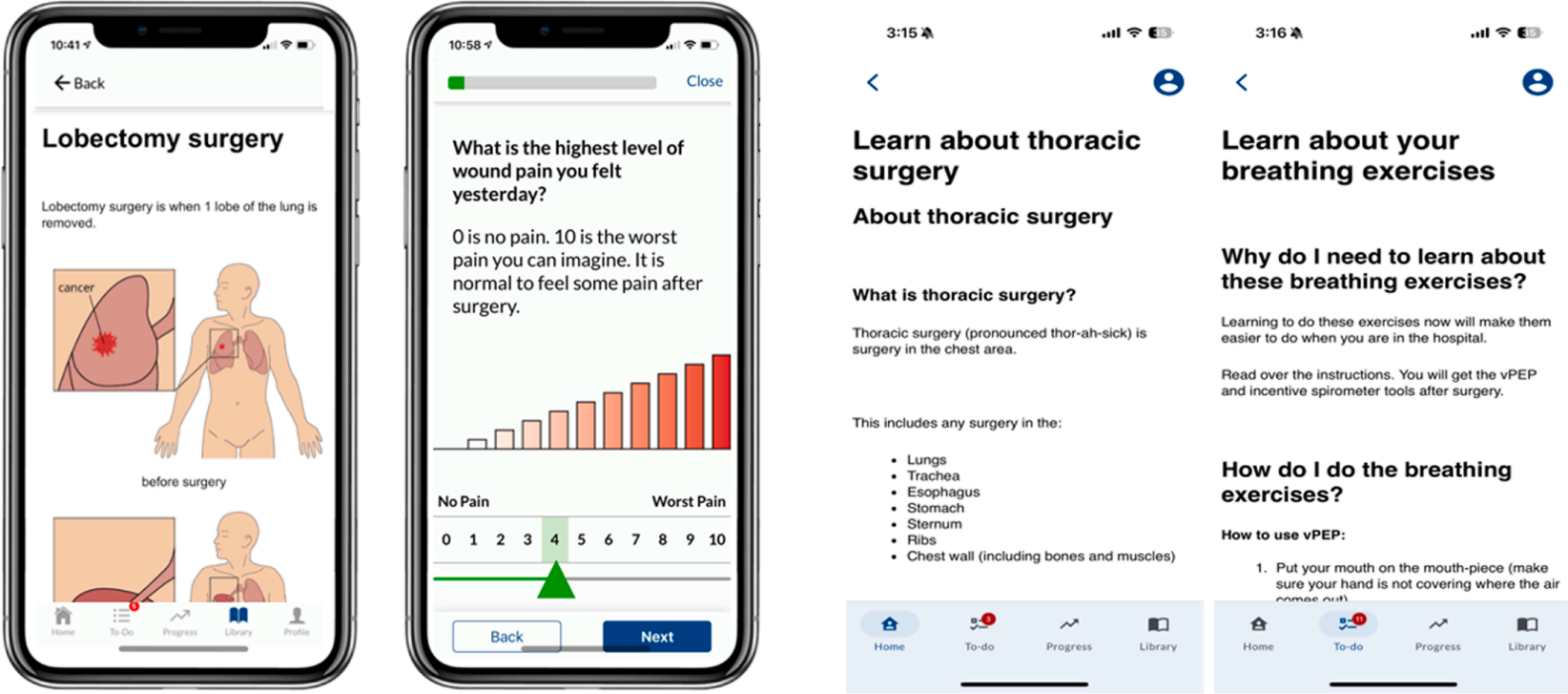
Examples of surveys, reminders, and educational material on the SeamlessMD platform.

Patients received health care reminders, educational content, health checks from day of activation until 30 days postoperatively, and surveys including the Patient-Reported Outcomes Measurement Information System Global-10 (PROMIS-10) and EuroQOL 5 Dimension (EQ-5) (Table E1, Table E2, Table E3, Table E4, Table E5 and Figure E1). Upon activation, the patient setup survey collected demographic information, including age, gender, smoking status, physical disability, and duration of time one could walk as well as three questions screening for health literacy. These questions come from the Short Test of Functional Health Literacy in Adults and are a validated measure to measure health literacy.^9^ Patients were stratified to adequate health literacy if they scored 4 or 5 on each question and inadequate healthy literacy if an answer to any question was ≤3. Patients who did not register and complete the initial setup survey were excluded.

Descriptive statistics were employed to evaluate PET utilization and initial observations using SPSS version 29 (IBM Corp). This study was approved by our institutional review board (IRB-300003564; approval date June 25, 2019).

## Results

Overall, 952 patients who underwent lung resection were enrolled in the study, of whom 88% (838 out of 952) activated the PET and completed the setup survey (Table 1). The PRO surveys PROMIS10 and EQ5 surveys were completed by 73% (613 out of 952) 15 days preoperatively, whereas only 17% (139 out of 952) of patients completed these surveys 30 days postoperatively. More than half (520 out of 952) of the cohort completed the “How you prepared for surgery” survey, whereas 37% (309 out of 952) completed at least 1 in-hospital health check. The 30-day health care utilization survey was completed by 39% (324 out of 952) of patients.

**Table 1:** Survey type and intervals completed

Survey	Description	Timing	No. Completed	% Compliance
Preoperative				
Set up survey	Sets patient characteristics	Day of enrollment	838	88
PROMIS10 + EQ-5	Validated surveys	15 d before surgery	613	73
Perioperative				
How you prepared for surgery	Compliance with preoperative instructions	Day of surgery	520	62
In-hospital health check	Daily check	Every day while in-hospital up to 14 d	309	37
After discharge				
Health care utilization	Survey about calls and visits to health care providers	30 d after discharge	324	39
PROMIS10 + EQ-5	Validated surveys	30 d after discharge	139	17

*PROMIS-10,* Patient-Reported Outcomes Measurement Information System Global-10; *EQ-5,* EuroQOL 5 Dimension.

Demographic information was obtained using the setup survey completed by 838 patients. The median age of this group was 65 years, women made up 52% (436 out of 838), and 68% (229 out of 838) had adequate health literacy (Table 2). Almost one-third (247 out of 838) were still smoking within a year of their operation. Seventeen percent (146 out of 838) reported some form of physical disability. Almost 20% (153 out of 838) were unable to walk more than 20 minutes at a time, whereas 35% (296 out of 838) could walk more than 20 minutes.

**Table 2:** Patient demographic characteristics (N = 838)

Demographic	Result
Age (y)	65 (57-72)
Gender	
Female	436 (52)
Male	402 (48)
Health literacy	
Adequate	229 (68)
Inadequate	106 (32)
Missing	183
Preoperative comorbidities	
Smoked within 1 y	247 (29)
Physical disability	146 (17)
Walking duration	
5 min	11 (1)
10-20 min	142 (17)
21-30 min	154 (18)
>30 min	142 (17)
Missing	389 (46)

Values are presented as n (%) or median (interquartile range).

The 30-day Healthcare Utilization survey found that of the patients who completed it, 32% (62 out of 196) made only 1 telephone call to the care team postoperatively, 16% (31 out of 196) made 2 telephone calls, and 10% (20 out of 196) made 3 or more telephone calls (Table 3). This same group reported using the PET allowed 10% (20 out of 196) to avoid only 1 telephone call, 14% (28 out of 196) to avoid 2 telephone calls, and 15% (29 out of 196) to avoid 3 or more telephone calls to the care team. Eleven percent of patients (22 out of 196) visited the ER only once and 1% (2 out of 196) visited twice. By using SeamlessMD, 5% (9 out of 196) of patients avoided only 1 ER visit, 2% (3 out of 196) avoided 2 visits, and another 2% (3 out of 196) avoided 3 visits to the ER. The readmission rate was 5% (10 out of 196). By using the PET, 91% (179 out of 196) of patients reported improved confidence in self-care and 86% (169 out of 196) felt less worried.

**Table 3:** Thirty-day health care utilization survey (n = 196)

Health care resource	Result
Telephone calls to health care team	
1	62 (32)
2	31 (16)
3+	20 (10)
Telephone calls avoided due to PET	
1	20 (10)
2	28 (14)
3+	29 (15)
ER visits	
1	22 (11)
2	2 (1)
ER visits avoided due to PET	
1	9 (5)
2	3 (2)
3	3 (2)
Readmissions	10 (5)
Improved confidence in self-care	179 (91)
Feel less worried	169 (86)

Values are presented as n (%). *PET*, Patient engagement technology; *ER*, emergency room.

Quality of life measures using PROMIS-10 and the EQ-5 surveys were completed by 120 patients 15-days preoperatively and 30-days postoperatively (Table 4). Trends observed in the PROMIS-10 survey over time show overall worse responses for most domains. Metrics were slightly worse for overall health, quality of life, physical health, mental health, pain, fatigue, and both social and physical activities, with pain, fatigue, and physical activity limitations being the most pronounced. Patients reported less emotional problems such as anxiety, depression, or irritability in the postoperative survey compared to preoperatively. Responses for the EQ-5 survey mirrored that of the PROMIS-10 because postoperative responses indicated more difficulty with physical activity, self-care, and pain, whereas anxiety and depression improved over the duration of the perioperative journey.

**Table 4:** Patient-Reported Outcomes Measurement Information System Global-10 and EuroQOL 5 Dimension patient-reported outcomes surveys completed at 15 days preoperatively and 30 days postoperatively (n = 120)

	15-d	30-d
In general, would you say your health is:		
1. Poor	4 (3)	2 (2)
2. Fair	15 (13)	15 (13)
3. Good	47 (39)	58 (48)
4. Very good	48 (40)	42 (35)
5. Excellent	6 (5)	3 (3)
In general, would you say your quality of life is		
1. Poor	3 (3)	1 (1)
2. Fair	8 (7)	15 (13)
3. Good	37 (31)	37 (31)
4. Very good	49 (41)	55 (46)
5. Excellent	23 (19)	12 (10)
In general, how would you rate your physical health?		
1. Poor	6 (5)	1 (1)
2. Fair	18 (15)	26 (22)
3. Good	53 (44)	56 (47)
4. Very good	38 (32)	34 (28)
5. Excellent	5 (4)	3 (3)
In general, would you rate your mental health including your mood and your ability to think?		
1. Poor	0 (0)	1 (1)
2. Fair	6 (5)	7 (6)
3. Good	42 (35)	45 (38)
4. Very good	46 (38)	47 (39)
5. Excellent	26 (22)	20 (17)
In general, how would you rate your satisfaction with your social activities and relationships?		
1. Poor	2 (2)	3 (3)
2. Fair	15 (13)	14 (12)
3. Good	30 (25)	41 (34)
4. Very good	47 (39)	45 (38)
5. Excellent	26 (22)	17 (14)
In general, please rate how well you carry out your usual social activities and roles. (This includes activities at home at work and in your community and responsibilities as a parent child spouse employee friend, etc.)		
1. Poor	3 (3)	4 (3)
2. Fair	11 (9)	19 (16)
3. Good	37 (31)	39 (33)
4. Very good	43 (36)	42 (35)
5. Excellent	26 (22)	16 (13)
To what extent are you able to carry out your everyday physical activities such as walking climbing stairs carrying groceries or moving a chair?		
1. Not at all	1 (1)	0 (0)
2. A little	17 (14)	14 (12)
3. Moderately	23 (19)	37 (31)
4. Mostly	16 (13)	32 (27)
5. Completely	63 (53)	37 (31)
In the past 7 days how often have you been bothered by emotional problems such as feeling anxious depressed or irritable?		
1. Always	2 (2)	1 (1)
2. Often	14 (12)	10 (8)
3. Sometimes	39 (33)	30 (25)
4. Rarely	45 (38)	38 (32)
5. Never	20 (17)	41 (34)
In the past 7 days how would you rate your fatigue on average?		
1. Very severe	0 (0)	0 (0)
2. Severe	7 (6)	6 (5)
3. Moderate	39 (33)	50 (42)
4. Mild	54 (45)	56 (47)
5. None	20 (17)	8 (7)
In the past 7 days how would you rate your pain on average?		
1. Pain score: 10	0 (0)	1 (1)
2. Pain score: 7, 8, or 9	9 (8)	8 (7)
3. Pain score: 4, 5, or 6	21 (18)	21 (18)
4. Pain score: 1, 2, or 3	47 (39)	67 (56)
5. Pain score: 0	43 (36)	23 (19)
Check 1 box that best describes your mobility today		
1. I have no problems in walking about	82 (68)	78 (65)
2. I have slight problems in walking about	13 (11)	26 (22)
3. I have moderate problems in walking about	23 (19)	14 (12)
4. I have severe problems in walking about	2 (2)	2 (2)
5. I am unable to walk about	0 (0)	0 (0)
Check 1 box that best describes your self-care today		
1. I have no problems washing or dressing myself	110 (92)	100 (83)
2. I have slight problems washing or dressing m	5 (4)	17 (14)
3. I have moderate problems washing or dressing	3 (3)	3 (3)
4. I have severe problems washing or dressing m	2 (2)	0 (0)
5. I am unable to wash or dress myself	0 (0)	0 (0)
Check 1 box that best describes your usual activities (eg, work study housework family or leisure)		
1. I have no problems doing my usual activities	69 (58)	46 (38)
2. I have slight problems doing my usual activities	27 (23)	46 (38)
3. I have moderate problems doing my usual acti	19 (16)	23 (19)
4. I have severe problems doing my usual activities	2 (2)	5 (4)
5. I am unable to do my usual activities	3 (3)	0 (0)
Check 1 box that best describes your pain/discomfort today		
1. I have no pain or discomfort	59 (49)	33 (28)
2. I have slight pain or discomfort	42 (35)	64 (53)
3. I have moderate pain or discomfort	16 (13)	20 (17)
4. I have severe pain or discomfort	3 (3)	3 (3)
5. I have extreme pain or discomfort	0 (0)	0 (0)
Check 1 box that best describes your anxiety/depression today		
1. I am not anxious or depressed	60 (50)	70 (58)
2. I am slightly anxious or depressed	46 (38)	39 (33)
3. I am moderately anxious or depressed	14 (12)	10 (8)

Values are presented as n (%).

## Discussion

In this retrospective cohort study, over 5 years, we examined survey completion rates, 30-day health care utilization, and PRO surveys among patients undergoing lung resection using a web-based app. Responses were collected in all stages of the perioperative journey up to 30 days after surgery. Patients reported that use of the app prevented more than 100 telephone calls to providers and 24 ER visits. This resulted in improved confidence in self-care and feeling less worried in >85%.

PROs can help establish patient expectations and may lead to improved outcomes. They are especially important in thoracic surgery where operations have longstanding influence on multiple components of well-being, namely dyspnea and functional status. Heiden and colleagues^1^ administered PROMIS surveys to patients undergoing surgery for non–small cell lung cancer over a 4-year period. They found pain and diminished physical function lingered for 6 months after surgery. Dyspnea related to surgery persisted for more than 1 year. This information is helpful when counseling patients on expectations with their recovery. The value of PROs has been established outside of thoracic surgery as well. In a randomized controlled trial at a single institution, patients receiving chemotherapy for metastatic solid tumors were dichotomized with 1 group receiving regular PRO surveys. The PRO group was found to have increased survival, median 31.2 months versus 26.0 months in those not sent PRO surveys.^10^ A proposed explanation is early recognition of negative sequalae related to chemotherapy, allowing providers to intervene. This supports the stance that insight into the patient experience during their perioperative journey should be considered equivalent to standard outcome measures.

Patient engagement technologies can help alleviate anxiety related to surgery and reduce health care utilization. Leading up to surgery, most patients are unsure what lies ahead, which can result in a feeling of helplessness. PETs can provide information to patients that allows more involvement in their own decision making and care. One study followed patients from multiple surgical subspecialties enrolled with a web-based app that provided education, recovery monitoring, and collection of PROs during their perioperative journey. Overall, >90% of patients reported the app helped them prepare for surgery.^11^ Much like our study, other works demonstrated high levels of patient-reported confidence in self-care.^8^^,^^11^, ^12^, ^13^ Literature suggests that patient involvement and understanding of their care can positively influence anxiety levels.^12^, ^13^, ^14^ Our study's finding that use of the PET reduced worry surrounding the perioperative journey adds to this observation. Patient empowerment has other benefits that extend beyond mental health. Smithson and colleagues^15^ reported that in the care for colorectal surgery patients, patient empowerment in self-care has been proven to result in earlier discharge after surgery. Through educational programs in the web-based apps, patients mastered the care of their ileostomy and left the hospital earlier than those who did not have access to the PET. This is an example of the influence that patient education delivered on a PET can on traditional quality metrics. Thus, further investigation into their potential benefits to thoracic surgery patients is needed. Use of PETs can also reduce health care utilization among patients. Multiple studies have shown 45% to 48% of patients avoided postoperative telephone calls and 13% to 28% avoided at least 1 ER visit by following instructions on a web-based app.^7^^,^^8^^,^^12^^,^^16^ This is congruent with our finding of >100 telephone calls to providers avoided and 24 ER visits avoided by using the PET.

Our work contributes to growing evidence that web-based apps are viable options for collecting patient reported outcomes while maintaining high satisfaction rates. There are several different options for survey administration, including in-person or remote and on paper or electronically. Prior work has shown that most patients prefer easy-to-use, convenient web-based apps that allow immediate feedback based on responses. Some patients also express that the app monitoring them remotely can be reassuring during their recovery from surgery.^2^ Khullar and colleagues^4^ were able to collect PROs from more than 3500 patients undergoing thoracic surgery during a 4-year period using tablets in clinic before transitioning to internet-based questionnaire links. Their overall survey completion rate was 65%.^4^ Heiden and colleagues^5^ studied implementation of PRO surveys using tablets in clinic. After multiple Plan-Do-Study-Act cycles, survey completion rates rose from 53% to 91%.^5^ In our study, we found high rates of patient satisfaction with the web-based app. One benefit is it allows patients to complete surveys in their own time. Our response rate significantly declined over time, which warrants more investigation. When choosing a mechanism of delivery, it is important to consider that many patients have geographic constraints, limited transportation, and financial hardships that prevent them from having adequate access to surgical services. Almost all adults in the United States own a cellular telephone, which can be used as a tool to bridge the access gap.^6^ Another potential benefit of the utilization of web-based apps is accuracy in data collection. A study comparing PROs in all stages of the perioperative journey found fewer errors in filling out forms on an electronic platform compared with classic pen and paper.^3^ Barriers exist to some patients activating and navigating PET platforms and thus creates a potential new disparity. Level of digital literacy, socioeconomic status, race, and gender have all been shown to influence rates of PET activation.^17^ These are important considerations when implementing PRO collection mechanisms.

There are several limitations to this study. It was conducted at 1 large academic medical center in the southeastern United States, and thus it is unclear if these findings are generalizable to other institutions, regions, or specialties. We included patients that self-selected in that they agreed to join the study, downloaded the app, and completed at least 1 survey. Use of the PET is only assessed through activation and completion of surveys because patients viewing educational materials without completing a survey could not be tracked. There was a significant rate of attrition with survey completion over time, which limits the generalizability of our findings. The use of a PET platform for this project may exclude vulnerable populations. This includes those who are unable to operate the application, do not have access to it due to lack of a smartphone or internet access, and those who are non-English speaking. Prior investigation of PET use by patients undergoing thoracic surgery at our institution found lower rates of survey completion among male patients, whereas those with adequate health literacy had higher survey completion rates.^18^ More work is needed to understand PET enrollment and survey compliance to ensure disparities can be eliminated, not propagated. Patient empowerment was noted as a reduction in health care utilization, but it is unclear if the appropriate medical decision was made by the patient because we did not collect this information.

## Conclusions

PETs provide a way to collect PROs. Among patients who utilize PETs in their perioperative care, there is a reported reduction in telephone calls to providers and ER visits, promotion of empowerment in self-care, and reduction of anxiety. Future work aimed at improving survey compliance is needed to ensure benefits of PETs are extended to all patients. The incorporation of PETs into the electronic medical record could be a way to ensure PRO data are collected alongside traditional metrics, which would provide a clearer picture of the patient perioperative experience.

### Webcast

You can watch a Webcast of this AATS meeting presentation by going to: https://www.aats.org/resources/impact-of-patient-engagement-t-9784.

**Figure:**
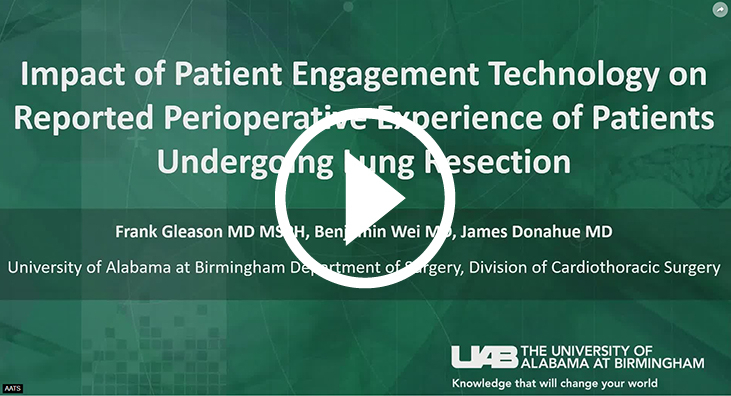

## Conflict of Interest Statement

The authors reported no conflicts of interest.

The *Journal* policy requires editors and reviewers to disclose conflicts of interest and to decline handling or reviewing manuscripts for which they may have a conflict of interest. The editors and reviewers of this article have no conflicts of interest.

## Figures and Tables

**Table E1:** Three-Question Brief Health Literacy tool, Patient Reported Outcomes Surveys (PROMIS-10), and EuroQOL 5 Dimension (EQ-5D)

3-Question Brief Health Literacy					
How confident are you in filling out medical forms by yourself?	Not at all (1)	A little bit (2)	Somewhat (3)	Quite a bit (4)	Extremely (5)
How often do you have problems learning about your medical condition because of difficulty understanding the written information?	Always (1)	Often (2)	Sometimes (3)	Occasionally (4)	Never (5)
How often do you have someone help you read hospital materials?	Always (1)	Often (2)	Sometimes (3)	Occasionally (4)	Never (5)
PROMIS Global-10 Score					
In general, would you say your health is:	Excellent (5)	Very Good (4)	Good (3)	Fair (2)	Poor (1)
In general, would you say your quality of life is:	Excellent (5)	Very Good (4)	Good (3)	Fair (2)	Poor (1)
In general, how would you rate your physical health?	Excellent (5)	Very Good (4)	Good (3)	Fair (2)	Poor (1)
In general, would you rate your mental health including your mood and your ability to think?	Excellent (5)	Very Good (4)	Good (3)	Fair (2)	Poor (1)
In general, how would you rate your satisfaction with your social activities and relationships?	Excellent (5)	Very Good (4)	Good (3)	Fair (2)	Poor (1)
In general, please rate how well you carry out your usual social activities and roles. (This includes activities at home at work and in your community and responsibilities as a parent child spouse employee friend etc.)	Excellent (5)	Very Good (4)	Good (3)	Fair (2)	Poor (1)
To what extent are you able to carry out your everyday physical activities such as walking climbing stairs carrying groceries or moving a chair?	Completely (5)	Mostly (4)	Moderately (3)	A little (2)	Not at all (1)
In the past 7 days how often have you been bothered by emotional problems such as feeling anxious depressed or irritable?	Never (5)	Rarely (4)	Sometimes (3)	Often (2)	Always (1)
In the past 7 days how would you rate your fatigue on average?	None (5)	Rarely (4)	Sometimes (3)	Often (2)	Always (1)
In the past 7 days how would you rate your pain on average?	No pain (0) – Worst pain imaginable (10)				
EQ-5D					
Check 1 box that best describes your mobility today	I am unable to walk about (5)	I have severe problems in walking about (4)	I have moderate problems in walking about (3)	I have slight problems in walking about (2)	I have no problems in walking about (1)
Check 1 box that best describes your self-care today	I am unable to wash or dress myself (5)	I have severe problems washing or dressing myself (4)	I have moderate problems washing or dressing myself (3)	I have slight problems washing or dressing myself (2)	I have no problems washing or dressing myself (1)
Check 1 box that best describes your usual activities (eg work study housework family or leisure)	I am unable to do my usual activities (5)	I have severe problems doing my usual activities (4)	I have moderate problems doing my usual activities (3)	I have slight problems doing my usual activities (2)	I have no problems doing my usual activities (1)
Check 1 box that best describes your pain/discomfort today	I have extreme pain or discomfort (5)	I have severe pain or discomfort (4)	I have moderate pain or discomfort (3)	I have slight pain or discomfort (2)	I have no pain or discomfort (1)
Check 1 box that best describes your anxiety/depression today	I am extremely anxious or depressed (5)	I am severely anxious or depressed (4)	I am moderately anxious or depressed (3)	I am slightly anxious or depressed (2)	I am not anxious or depressed (1)

**Table E2:** Set up survey

What is your gender?	Male Female				
Have you been feeling anxious in the past 24 hours?	0 (no anxiety) – 10 (maximum)				
Have you used tobacco within the last year?	Yes No				
Have you used drugs within the last year?	Yes No				
Do you have diabetes?	Yes No Not Sure				
Do you use anti-inflammatory medications?	Yes No				
Do you have a physical disability tat makes it hard for you to walk?	Yes No				
How confident are you in filling out medical forms by yourself?	Not at all (1)	A little bit (2)	Somewhat (3)	Quite a bit (4)	Extremely (5)
How often do you have problems learning about your medical condition because of difficulty understanding the written information?	Always (1)	Often (2)	Sometimes (3)	Occasionally (4)	Never (5)
How often do you have someone help you read hospital materials?	Always (1)	Often (2)	Sometimes (3)	Occasionally (4)	Never (5)
How do you prefer to receive medical information?	On websites In smartphone apps In videos In print Through emails Through text messages Explained over the phone Explained face-to-face				
How were you told about SeamlessMD?	Explained briefly Explained in detail Received email Received information sheet				
Do you need assistance managing your SeamlessMD account?	Free text response				

**Table E3:** How you prepared for surgery survey

At your preoperative assessment clinic (PAC) appointment did someone teach you how to prepare for surgery?	Yes print and verbal instructions Yes print instructions only Yes verbal instructions only No instructions
At your preoperative assessment clinic (PAC) appointment did someone teach you what to expect during your hospital stay?	Yes print and verbal instructions Yes print instructions only Yes verbal instructions only No instructions
Did you have any trouble getting to the hospital?	Yes No
Did you exercise regularly before surgery?	Exercise 1 to 2 times a week Exercise 3 to 5 times a week Exercise more than 5 times a week No exercise
What made it hard for you to exercise?	Free text
Did you learn about your breathing exercises before surgery?	Yes No
What made it hard for you to learn your exercises before surgery?	Free text
Did you stop smoking before surgery?	Yes No N/A
What made it hard for you to stop smoking before surgery?	Free Text
Did you stop taking anticoagulants (blood thinners) before surgery?	Yes No N/A
What made it hard for you to stop taking anti-coagulants (blood thinners) before surgery?	Free text
Did you stop taking anti-platelets (blood thinners) before surgery?	Yes No N/A
What made it hard for you to stop taking anti-platelets (blood thinners) before surgery?	Free text response
Did you stop taking all vitamins minerals and herbal products 1 week before surgery?	Yes No N/A
What made it hard for you to stop taking all vitamins minerals and herbal products?	Free text response
Did you stop drinking alcohol 2 days before surgery?	Yes No N/A
What made it hard for you to stop drinking alcohol before surgery?	Free text response
Did not shower before surgery with germ killing soap (chg soap)	Yes No N/A
What made it hard for you to take 3 showers using germ killing soap (chg soap)?	Free text response
Did you watch Emmi® videos?	Watched both Emmi videos Watched 1 Emmi video Did not watch Emmi videos
What made it hard for you to watch the Emmi® videos?	Free text response
Did you stop eating any food midnight the day before surgery?	Yes No N/A
What made it hard for you to stop eating after midnight the day before surgery?	Free text response
Did you stop drinking clear liquids at 5 am on the day of your surgery?	Yes No N/A
What made it hard for you to stop drinking clear liquids before surgery?	Free text response

**Table E4:** In-hospital health check

What is the highest level of pain you felt yesterday?	0 (no pain) – 10 (maximum)
How much nausea did you feel yesterday after surgery?	0 (no nausea) – 10 (maximum)
Is your pain well controlled with pills?	Yes No
How much time did you spend out of bed yesterday after surgery?	0 hours 1 to 2 hours 3 to 4 hours 5 to 6 hours 7 to 8 hours 9 to 10 hours 11 hours or more
How many walks did you take yesterday?	0 walks 1 walk 2 walks 3 walks 4 or more walks
What made it hard for you to take walks yesterday?	Free text response
What made it hard for you to do your exercises yesterday?	Free text response
Did you eat solid food yesterday?	Yes No
Did you drink liquids yesterday?	Yes No
Did you pass gas (fart) yesterday?	Yes No
Have your IV fluids been turned off?	Yes No N/A
Did you brush your teeth 2 times yesterday?	Yes No
What made it hard to brush your teeth 2 times yesterday?	Free text response
Are you worried about taking care of yourself after going home?	No I am not worried Yes a little Yes a lot
Is there anything else your hospital team should know?	Free text response

*N/A*, Not available.

**Table E5:** Health care utilization

In the 30 days since you left the hospital how many times have you visited your family doctor?	0 times 1 time 2 times 3 or more times
In the 30 days since you left the hospital how many times have you called your hospital health care team?	0 times 1 time 2 times 3 or more times
In the 30 days since you left the hospital how many calls to your hospital health care team has this online program helped you avoid?	0 times 1 time 2 times 3 or more times
What questions would you have called to ask about?	Free text response
In the 30 days since you left the hospital how many times have you visited an emergency room?	0 times 1 time 2 times 3 or more times
In the 30 days since you left the hospital how many visits to an emergency room did this online program help you avoid?	0 times 1 time 2 times 3 or more times
What problems would you have gone to an emergency room for?	Free text response
In the 30 days since you left the hospital how many times have you had to go back and stay overnight at the hospital?	0 times 1 time 2 times 3 or more times
In the 30 days since you left the hospital how many days have you stayed overnight at the hospital?	0 times 1 time 2 times 3 or more times
In the 30 days since you left the hospital did this online program help you feel more confident about taking care of yourself at-home?	No not much Yes a little Yes a lot
In the 30 days since you left the hospital did this online program help you feel less worried?	No not much Yes a little Yes a lot
What do you like the most about this online program?	Free text response
How can we make this online program more helpful for patients?	Free text response
Did you have any technical problems while using the online program?	Free text response
Do you have any other thoughts or comments to share with us?	Free text response
